# Supplementary figures and images for: Laterodorsal tegmentum–ventral tegmental area projections encode positive reinforcement signals
Source: J Neurosci Res. 2021 Aug 10;99(11):3084–100. doi: 10.1002/jnr.24931 (PMC9541203; doi:10.1002/jnr.24931)

# Sup. Figure 1

a

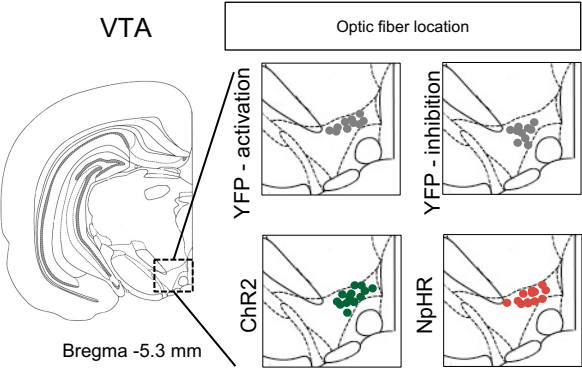

## Sup. Figure 2

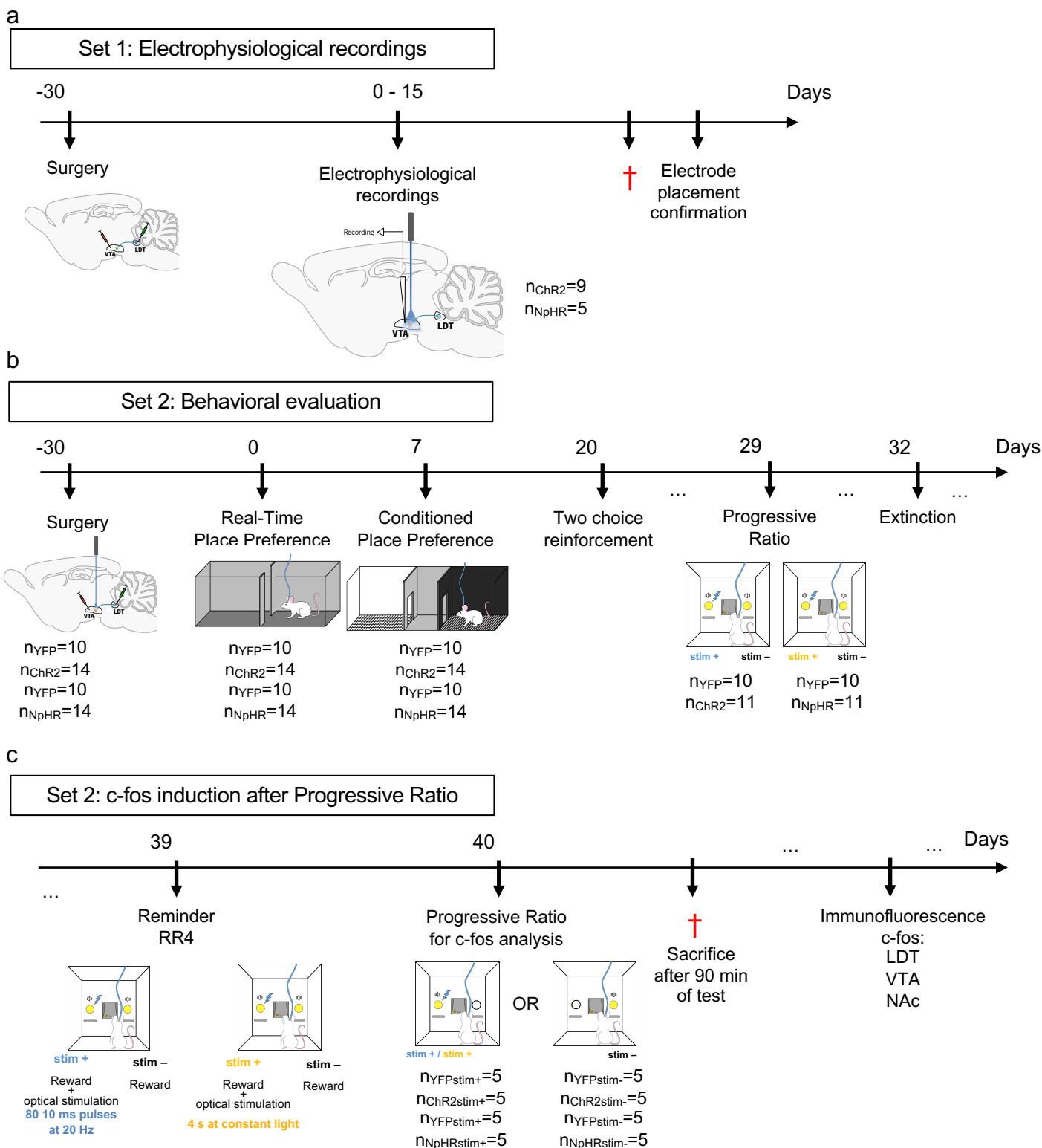

# Sup. Figure 3

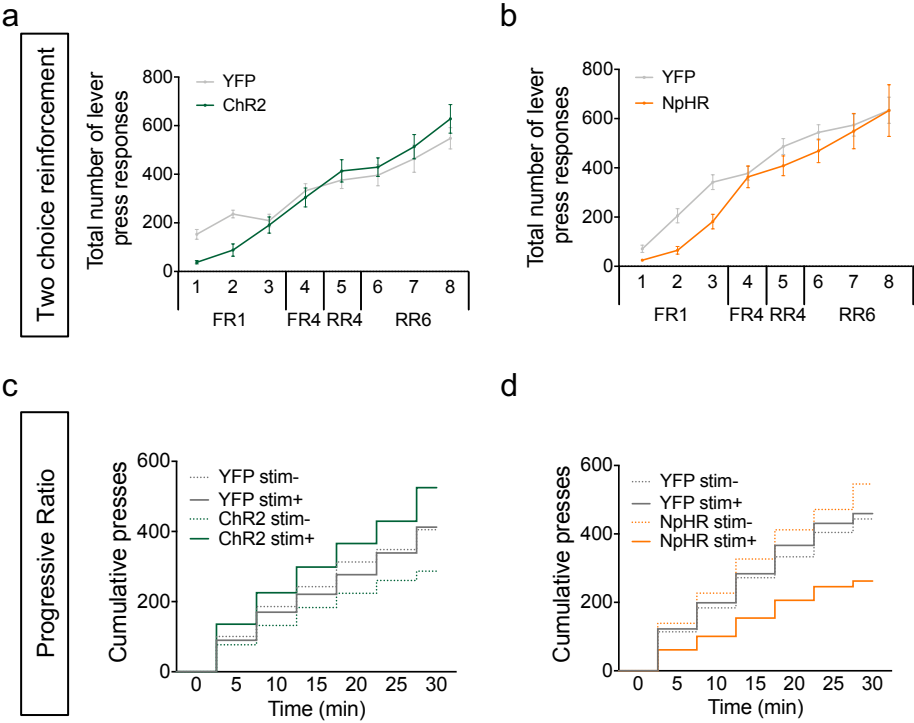

Sup. Figure 4

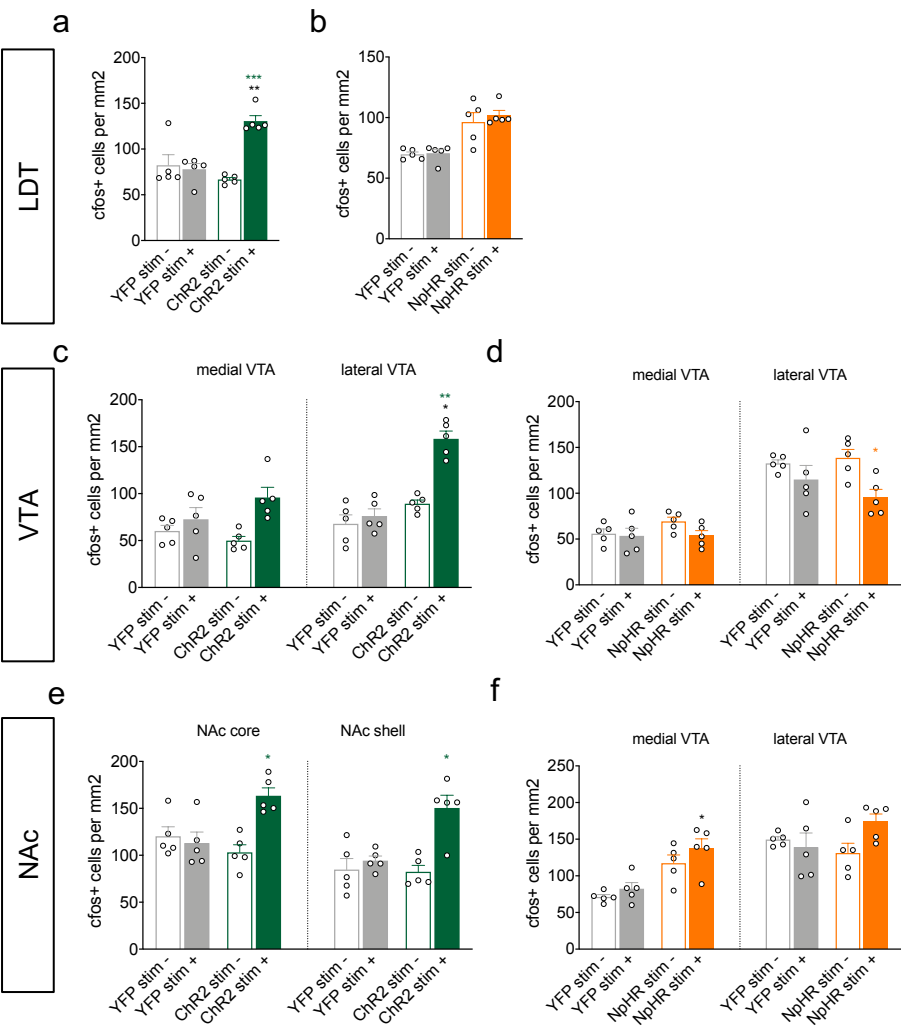

Sup. Figure 5

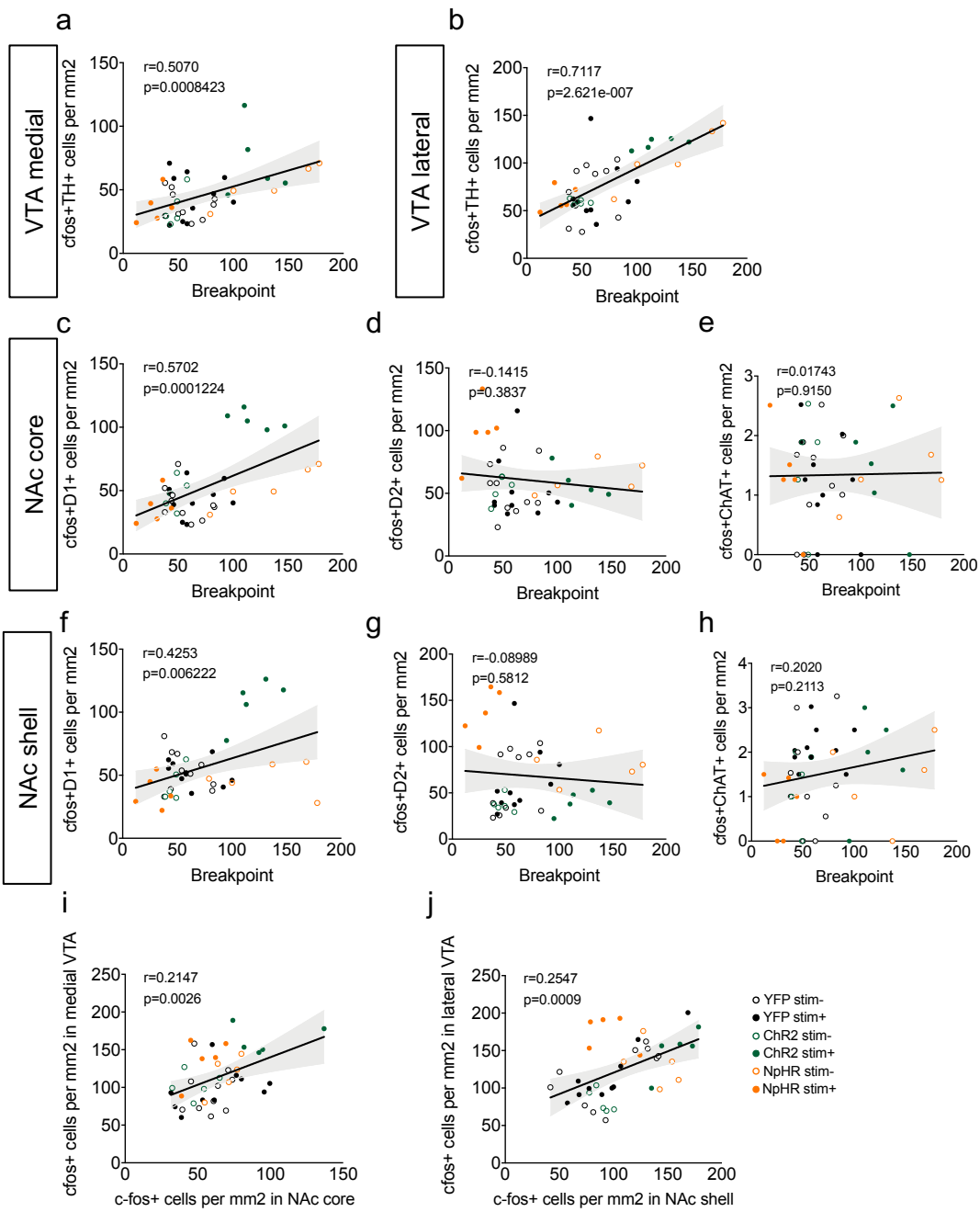

Supplement: Supplementary file 3 — FIGURE S1 Confirmation of optic fiber implantation for behavioral experiments. (a) Optic fiber placement in the VTA for YFP (activation—Stimulation with blue laser), ChR2, YFP (inhibition—Stimulation with yellow laser) and NpHR animals FIGURE S2 Experimental design for electrophysiological and behavioral studies. (a) For set 1 of experiments, for electrophysiological recordings, 30 days after surgery for virus injections in LDT and VTA, animals were subjected to anesthesia in order to place a recording electrode together with an optic fiber in VTA, to stimulate LDT terminals. Confirmation of electrode placement was confirmed after the experiment. (b) For behavioral experiments, 30 days after surgery for virus injections in LDT and VTA, and cannula implantation in the VTA (n of animals indicated in figure), animals from all four groups of set 2 performed the real‐time place preference (1 day). In the following week, the same cohort performed the conditioned place preference (3 days), Operant behavior in the two‐choice schedule of reinforcement started on the 20th day where animals are able to choose between pressing a lever for a reward and optical stimulation (stim+) or another lever for a reward alone (stim−), with increasing effort, until a random ratio 6 (RR6). This is followed by two sessions of a progressive ratio (PR) task for each lever. Then, animals performed the extinction phase of the two‐choice reinforcement behavior, where the reward was omitted for both levers but optical stimulation remained for the stim+ lever, for 4 days. For operant behavior the number of animals is reduced, since we had to exclude animals that lost the fiber implant. (c) Experimental design for c‐fos induction. After the two‐choice reinforcement operant protocol, animals performed a reminder session and, on the next day, followed with a PR session for c‐fos activation studies. In order to distinguish cell recruitment for task performance for a pellet alone or a pellet associated w [file JNR-99-3084-s003.pdf]
